# Prostate Cancer Characteristics in Renal Transplant Recipients: A 25-Year Experience From a Single Centre

**DOI:** 10.3389/fsurg.2021.716861

**Published:** 2021-07-29

**Authors:** Pietro Spatafora, Francesco Sessa, Simone Caroassai Grisanti, Claudio Bisegna, Calogero Saieva, Giandomenico Roviello, Paolo Polverino, Anna Rivetti, Lorenzo Verdelli, Maria Zanazzi, Detti Beatrice, Graziano Vignolini, Gabriella Nesi, Giulio Nicita, Sergio Serni, Donata Villari

**Affiliations:** ^1^Unit of Urological Robotic Surgery and Renal Transplantation, Careggi Hospital, Florence, Italy; ^2^Department of Experimental and Clinical Medicine, University of Florence, Florence, Italy; ^3^Cancer Risk Factors and LifeStyle Epidemiology Unit, Cancer Research and Prevention Institute—Istituto per lo Studio e la Prevenzione Oncologica (ISPO), Florence, Italy; ^4^Department of Medical Oncology, Careggi Hospital, Florence, Italy; ^5^Department of Nephrology, Dialysis and Renal Transplantation, Careggi Hospital, Florence, Italy; ^6^Department of Radiotherapy, Careggi Hospital, Florence, Italy; ^7^Division of Pathological Anatomy, University of Florence, Florence, Italy

**Keywords:** renal transplantation, prostate cancer, prostatectomy, radiotherapy, immunosuppression, post-transplant malignancies

## Abstract

**Objectives:** The incidence of prostate cancer in renal transplant recipients (RTRs) is increasing, but few data are available in the literature. In this study, we reviewed the 25-year experience in the management of prostate cancer after kidney transplantation at the Florence Transplant Centre.

**Methods:** We retrospectively reviewed the data from 617 RTR male patients who underwent renal transplantation at our institute between July 1996 and September 2016. Data regarding demographics, renal transplantation, prostate cancer and immunosuppressive treatment were analyzed. The probability of death was estimated by using the Kaplan-Meier method and differences between patients' groups were assessed by the log-rank test.

**Results:** From July 1991 to September 2016, 617 kidney transplantations of male patients were performed at our institute. Among these, 20 patients were subsequently diagnosed with prostate cancer accounting for a cumulative incidence of 3.24%. After a median follow-up of 59 months, 10 patients underwent radical prostatectomy whereas 10 patients underwent primary radiotherapy. A biochemical recurrence was identified in five (25%) patients while a fatal event occurred in 11 (55%) patients. Univariate Cox regression showed that the basal value of PSA >10 ng/ml was the only significant factor negatively affecting the survival of patients.

**Conclusions:** Standard treatments can be proposed to RTR with satisfactory results on both post-operative and oncological outcomes. Further studies are needed to address the issue of prostate cancer screening based on PSA levels and the optimal management of prostate cancer in RTRs.

## Introduction

Kidney transplantation is considered the standard of care for patients with end-stage kidney disease under chronic dialysis treatment (1). Today, modern surgical techniques have dramatically improved the quality of life and the overall survival of renal transplant recipients (RTRs) (1). Besides, the use of novel immunosuppressors have increased the 1-year graft survival rate and decreased acute rejection rate (2). Unfortunately, several transplantation-related diseases including cancer, cardiovascular disease and infection may affect the survival of renal transplant recipients. It has been estimated that RTRs are two- to five-fold more likely to develop cancer compared to the general population (3, 4). Therefore, the development of cancer has become a major concern as it is currently one of the main causes of death in RTRs. The increasing incidence of post-transplant malignancies is generally attributed to immunosuppression which leads to impaired immunosurveillance of cancer cells and viral infections capable of cancer development. Additionally, it has been observed a direct and specific pro-oncogenic effect on RTRs of immunosuppressive drugs (5, 6) and other immunosuppression-independent factors such as the increased age of RTRs, the male gender and the pre-transplant dialysis duration (7, 8).

Prostate cancer (PCa) is the second most diagnosed cancer in men and the most common non-skin solid neoplasm in RTRs (9, 10). Nevertheless, the real incidence of PCa in RTR remains controversial; in fact, on one hand RTRs are screened less frequently following transplantation than men in the general population, so that some cancers are missed, on the other immunosuppressive therapy (including azathioprine and calcineurin inhibitors) are associated with an increased risk of cancer and enhanced *in vitro* and *in vivo* PCa aggressiveness (11, 12). Generally, the vast majority of post kidney transplantation prostate cancers are localized; however, due to the lack of randomized studies, no specific guidelines for the management of localized prostate cancer are available and, consequently, RTR patients are being treated with surgery or radiotherapy according to national or local guidelines (13). Nonetheless, treatment modalities for RTR include radical prostatectomy (RP), radiation therapy (RT), hormonal therapy and, recently, active surveillance (AS). Moreover, the concomitant use of immunosuppressors as well as the presence of the kidney graft in the pelvis make the treatment for localized PCa after kidney transplantation (KT) more challenging, due to an increased postoperative morbidity and higher rate of tumor progression given their vulnerability to infection due to immunodeficiency and the oncogenic status of immunosuppressive treatments. Additionally, the surgery in RTR is complicated due to several factors, including the distortion of normal tissue planes, the pelvic location of the transplant kidney with difficulty to perform bilateral lymphadenectomy (14, 15). Conversely, RT might be associated with risk of allograft injury, ureteral injury and urethral stricture (16, 17).

Of note, there are not standardized guidelines for screening or management of PCa in RTRs, suggesting an urgent need for structured and well-designed address to urological centres expirienced in both oncological and transplant surgery.

Aim of this study was to review a 25-year experience at the Florence Transplant Center in the management of PCa after KT, focusing on treatment and oncological outcomes.

## Method

### Patients

We retrospectively reviewed data from all male patients who underwent renal transplantation at our institute between July 1991 and September 2016, identifying those who developed PCa during the scheduled follow-up. Patients' information on demographics and characteristics of newly diagnosed prostate cancer were collected.

### Diagnosis and Staging

All male > 40 years candidates to renal transplantation were previously screened for prostate-specific antigen (PSA) and digital rectal exploration (DRE). Indications for prostate biopsy in both KT candidates and patients during follow-up were PSA > 4 ng/ml and/or suspicious DRE. If indicated, patients underwent transrectal prostate biopsy with ultrasound guidance, according to standard guidelines (11). All PCa were biopsy-proven.

### Prostate Cancer Treatment and Follow-Up

Once the diagnosis was established, cancer staging was determined according to the 8th Edition Prostate Cancer Staging by the American Joint Committee on Cancer. Staging of disease was completed with abdomen or bone CT scan when clinically required (13, 18, 19). We assessed the class of risk using EAU/D'Amico's score (13). The study was approved by the local Ethical Committee and all patients provided written informed consent.

Radical prostatectomy was performed by open approach. A pelvic bilateral lymphadenectomy was adopted in selected cases.

Radiation therapy (RT) was delivered with a total dose ranging from 78 to 80 Gy in patients with definitive RT and from 66 to 72 Gy in patients with adjuvant/salvage RT, normally applied as 2 Gy single fraction. For patients requiring RT as primary treatment, androgen deprivation therapy (ADT) consisted of 3 years of LHRH analog (leuprolide, triptorelin or goserelin) administration.

Follow-up after primary treatment included medical examination and PSA dosage every 3 months for the first 2 years and every 6 months after 2 years, according to the available evidence (13). Biochemical failure was defined as either a two-time PSA rise of 0.2 ng/ml, a PSA rise above 2 ng/ml for patients who underwent radiotherapy or a PSA doubling time <6 months, according to AUA guidelines (20).

### Survival Analyses

The probability of death was estimated by using the Kaplan-Meier method and differences between patients' groups were assessed by the log-rank test. The time of observation was calculated from the date of biopsy until the date of death or last follow-up for patients still alive (December 31, 2019). The event of interest was occurrence of death for all causes (Overall Survival). The estimated relative risk of death was expressed as hazard ratios (HR) and corresponding 95% confidence interval (95% CI). Univariate Cox regression models were used to evaluate the effect of each specific parameter. All statistical tests were two-sided, and *p*-values <0.05 were considered statistically significant. All statistical analyses were performed by SPSS (Statistical Package for Social Science, version 22) software.

## Results

From July 1991 to September 2016, 617 kidney transplantations of male patients were performed at our institute. Among these, considering patients >40 years undergoing PCa screening, 20 patients with a subsequent diagnosis of PCa were identified (cumulative incidence of 3.84%). The characteristics of patients undergoing KT for all 20 patients are described in the first half of Table 1. The main cause of renal disease that led to transplantation was glomerulonephritis, diagnosed in 8 (40%) patients after a median time for dialysis of 30 months (range, 3–192 months). The median age at transplantation was 55 years (range, 36–71 years). One patient required secondary renal transplantation. All patients were on immunosuppressive therapy (IS) maintenance, according to our standard protocol: double IS was administered to 4 patients based on calcineurin inhibitors (CNI) and steroids; triple IS was administered in association with Azathioprine (AZA) to three patients, in association with Mycophenolate Mofetil (MMF) to 12 patients and in association with mTOR inhibitors (Everolimus) to one patient.

**Table 1:** Characteristics of the study participants.

**Recipient variables**	**PCa in RTR**
	***n* = 20**
**Age at renal transplantation**	
mean ± SD (range), years	53.7 ± 9.5 (36.1–70.8)
**Age at PCa diagnosis**	
mean ± SD (range), years	63.3 ± 6.4 (50.0–74.7)
**Primary renal disease leading to RTR**, ***n*****(%)**	
Glomerulonephritis	8 (40)
Chronic pyelonephritis/tubulointerstitial disease	3 (15)
Autosomal dominant polycystic kidney disease	2 (10)
Chronic kidney disease—not specified	4 (20)
Diabetic nephropathy	2 (10)
Other specified etiologies	1 (5)
**Time on dialysis**	
mean ± SD (range), months	41.85 ± 41.66 (3–192)
**Comorbidities**, ***n*****(%)**	
Arterial hypertension	13 (65)
Diabetes mellitus type 2	2 (10)
Dyslipidemia	8 (40)
Obesity	6 (30)
Metabolic syndrome	3 (15)
**Baseline immunosuppression**, ***n*****(%)**	
calcineurin inhibitors and steroids	4 (20)
calcineurin inhibitors, steroids and azathioprine	3 (15)
calcineurin inhibitors, steroids and mycophenolate Mofetil	12 (60)
calcineurin inhibitors, steroids and mTOR-I	1 (5)
**Other cancers diagnosed before PCa**, ***n*****(%)**	
Yes	8 (40)
Nonmelanoma skin cancer	4
Adenocarcinoma of pancreas	1
Adenocarcinoma of thyroid	1
Squamous cell carcinoma of lung	1
Post-transplant lymphoproliferative disorder	1
No	12 (60)
**Type of donors**, ***n*****(%)**	
Cadaveric	20 (100)
Live	0 (0)
**PSA**, ***n*****(%)**	
<10 ng/ml	17 (85)
>10 ng/ml	3 (15)
**Gleason score at transrectal ultrasound biopsy**, ***n*****(%)**	
GS 6 (3 + 3)	8 (40)
GS 7 (3 + 4)	4 (20)
GS 7 (4 + 3)	3 (15)
GS 8 (4 + 4)	3 (15)
GS 9 (4 + 5)	2 (10)
**Clinically localized prostate cancer**, ***n*****(%)**	20 (100)
**Clinically advanced prostate cancer**, ***n*****(%)**	0 (0)
**Gleason score, pT stage after open radical prostatectomy**, ***n*****(%)**	10 (100)
GS 6 (3+3), pT2c, R0	5 (50)
GS 7 (3+4), pT2c, R0	2 (20)
GS 8 (4+4), pT3b, R0	1 (10)
GS 9 (4+5), pT3b, R0	2 (20)
**Time between renal transplantation**	
and PCa diagnosis, median (range), years	9.6 **±** 7.4 (0.125.4)

*PCa, prostate cancer; RTR, renal transplant recipients; SD, standard deviation; PSA, prostate specific antigen; RT, radiotherapy; RP, radical prostatectomy; GS, Gleason score*.

The median age at diagnosis of PCa was 64 years (range, 52–74 years) with median PSA value of 6.9 ng/ml (range, 2.9–16 ng/ml). A single patient (5%) reported a PSA value of <4 ng/ml. In this case the indication to prostate biopsy was supported by a suspicion at DRE. Three patients came to our attention with a first relief of PSA > 10 ng/ml after KT received years before. No evidence of metastasis or lymph node involvement was found at diagnosis at preoperative imaging (Table 1).

Primary treatment of PCa is summarized in Table 2. Overall, 10 (50%) patients underwent open retropubic radical prostatectomy. No patients underwent active surveillance or brachytherapy. Gleason score was higher than 7 for 3/10 (15%) patients. Bilateral lymphadenectomy was performed in 5 cases (25%, pN0 in four cases, pN1 in one case) and median lymph nodes removed was 13 (IQR 9–17), unilateral lymphadenectomy was performed in 5 cases (25%) and median lymph nodes removed was 7 (IQR 4–9). Primary treatment with radiotherapy was performed in 10 (50%) patients.

**Table 2:** Characteristics of the primary treatment.

**Characteristics**	**PCa in RTR**
	***n* = 20**
**Primary treatment**	
Surgery, Open retropubic RP, *n* (%)	10 (50)
Lymphadenectomy, *n* (%)	10 (50)
Monolateral, *n* (%)	5 (25)
Bilateral, *n* (%)	5 (25)
RT, *n* (%)	10 (50)
**Complications**, ***n*****(%)**	
Requiring endoscopic treatment	2 (10)

*PCa, prostate cancer; RTR, renal transplant recipients; RP, radical prostatectomy; RT, radiotherapy*.

Post-operative PSA was <0.01 1 months after surgery in eight patients, while in two patients was > 0.5 ng/ml; in case of RT as primary treatment, PSA post treatment was in all cases <2 ng/ml.

Three patients undergoing surgery experienced relapse of PCa and additional treatments were required. In particular, in one case a salvage radiotherapy treatment for biochemical recurrence at a median time of 6 months after surgery was performed. The remaining two patients reported post-operative PSA > 0,5 ng/ml (pT3b, GS 4 + 5 = 9), developed bone metastasis at bone scintigraphy 6 months after surgery and were scheduled for local RT + ADT.

After a median follow-up of 59 months (range, 3–131 months), biochemical recurrence occurred in five (25%) patients. Moreover, median eGFR was 47 ml/min/1.73m^2^ (IQR 33-69) and no graft loss was observed although renal function deterioration was reported by one patient. Eight (40%) patients developed a secondary tumor, mainly skin squamous carcinoma. For 11 (55%) patients with fatal event (not related to prostate cancer disease), univariate Cox regression showed that the basal value of PSA >10 ng/ml was the only statistically significant factor influencing the overall survival of patients. The Cox regression analysis failed to highlight any other potential predictor of survival (Table 3).

**Table 3:** Kaplan Meier overall survival (OS) analysis of 20 PCa cases after kidney transplantation: patients at start, events (deaths), *p*-value from log rank test, hazard ratios with 95% confidence intervals from cox regression analysis.

	**Overall survival**			**Cox regression**	
**Characteristic**	**N** ^**◦**^ **at start**	**N** ^**◦**^ **deaths**	***P*-value** ^*^	**HR (95%CI)**	***P*** ^**◦**^
**Age at transplantion (ys)**					
≤ 55	10	4	0.27		
> 55	10	7			
**Age at cancer diagnosis (ys)**					
≤ 64	10	3	0.09		
> 64	10	8			
**Transplant-cancer interval (ys)**					
≤ 8.6	10	6	0.47		
> 8.6	10	5			
**PSA at diagnosis (ng/ml)**					
<10	17	8		1	
>10	3	3	**0.029**	**4.25 (1.04–17.4)**	**0.044**
**Bioptic gleason score**					
≤7	15	7	0.43		
>7	5	4			
**Pathologic gleason score**					
≤7	7	2	0.35		
>7	3	1			
**cT stage**					
2	12	6	0.82		
3	8	5			
**Primary therapy**					
Surgery	10	3	0.061		
RT	10	8			
**Hormonotherapy**					
No	18	9	0.41		
Yes	2	2			
**Post-surgery RT**					
No	17	8	0.59		
Yes	3	3			
**Other cancers**					
No	9	4	0.71		
Yes	11	7			
**Biochemical recurrence**					
No	17	9	0.79		
Yes	3	2			
**Immunosuppresive therapy**					
Other	17	9			
Cyclosporine+steroid+azathioprine	3	2	0.24		
**Gleason Score group at transrectal ultrasound biopsy**					
GS 6 (3 + 3)	8	6			
GS 7 (3 + 4)	4	0	0.49		
GS 7 (4 + 3)	3	1			
GS 8 (4 + 4)	3	3			
GS 9 (4 + 5)	2	1			
**Gleason Score group after open radical prostatectomy**					
GS 6 (3 + 3)	5	1			
GS 7 (3 + 4)	2	1	0.34		
GS 8 (4 + 4)	1	0			
GS 9 (4 + 5)	2	1			
**Total**	**20**	**11**			

*Ys, years; RT, radiotherapy; GS, Gleason score*.

* *P-value from log rank test*;

◦ *p-value from Cox regression analysis*.

*They represent the Hazard Ratio (HR), the 95% Confidence Interval (CI) and the p value from Cox regression analysis*.

## Discussion

Nearly 37.000 new cases of PCa are expected in Italy in 2019. PCa is the most diagnosed cancer (19%) and the third cause of death within the male Italian population (8%). A north-south gradient of incidence is evident with higher standardized rates in the North-Italy (145.1/100,000) than in the Center (137.6, −5%) or South-Italy (106.6, −27%) (21). In Tuscany, PCa is the most diagnosed malignancy (20%) according to the most recent cancer statistics reviews released in 2020. Around 2,200 new cases of PCa are diagnosed every year, which include ~650 cases in the city of Florence (22). In our study, we retrospectively evaluated 20 patients with PCa diagnosed after kidney transplantation. Albeit the retrospective nature of data and the limited number of patients, we observed that PCa in RTRs showed outcomes in line with those of population with PCa treated with curative treatment. Univariate Cox regression showed that the basal value of PSA >10 ng/ml was the only significant factor negatively affecting the survival of patients. In 2018, a systematic review of the European Association of Urology on Renal Transplantation Guidelines panel including 41 studies and 319 RTR patients diagnosed with localized PCa, showed that oncological outcomes are similar to the general population when performed at referral centres (9). In line with these data, our study confirms and corroborates the importance of treating PCa patients who underwent kidney transplantation at highly specialized centres.

To date, the role of screening for PCa has been somewhat controversial. The US Preventive Services Task Force does not recommend age-indiscriminate routine PSA-based screening for men, according to the European Randomized study for Screening of Prostate Cancer (ERSPC) trials, which reported a borderline reduction in PCa mortality (23). On the contrary, The American Urological Association recommends routine PSA-based screening for men in the highest risk age group (55–69 years) only (24). However, the vast majority of analogous epidemiological studies were carried out with data obtained before the introduction of PSA-based screening (25); as a consequence, epidemiological data have become increasingly heterogeneous, with several discrepancies based on screening protocols and/or national/local guidelines (25). Additionally, no robust evidence on both duration and modalities of PSA screening are available nowadays in this clinical context and, as consequence, findings on diagnosis as well as treatment of PCa might be misleading. In this regard, Becher et al. reported in their experience that (PSA) appears to be as reliable for PCa screening of transplant candidates and recipients as it is for the general population (26). Conversely, The ASM (American Society of Nephrology) and the AST (American Society of Transplantation) recommended that all RTRs at age 50 years with a life expectancy of 10 years or more should be monitored and screened for prostate cancer before and after transplantation. Any prostate cancer screening program in renal transplant candidates should be based on a mortality risk model. It is established that the efficacy of any screening program in terms of improvement in cancer specific mortality should be evaluated beyond a 10-year horizon (27–29). Many studies investigating PCa in RTRs are epidemiological or descriptive and based on a limited number of patients. Given this background, the utility of PCa screening, performed in all candidates to renal transplantation to ensure appropriate kidney allocation, remains still debated. Bratt et al. in their national renal register and PCa register identified 133 KT recipients, transplantated before 1990s when PSA testing became common, and concluded their analysis that immunosuppression after KT did not increase the risk of PCa or adversely affected PCa outcomes (30). In our study, PCa incidence for RTRs was 3.24%, slightly higher when compared to previously published reports where incidence ranged from 0.3 to 2% (7, 31). Although RTRs are three times more likely to develop malignancies than the general population, there is no evidence of a greater risk for developing PCa. Both European and American guidelines recommend routine PSA-based screening for all PCa transplant candidates starting at age 50, although their studies observed similar or reduced risk of PCa compared to the general population (32).

In our study, the median time between renal transplantation and PCa diagnosis was 107 months and, in line with these data, other studies reported a similar time interval between kidney transplantation and PCa development (122 months) (25), supporting the idea that PCa in RTRs should not always be considered an early event. Moreover, we observed a time interval of approximately 10 years between the median age at transplantation (55 years) and the median age at the time of PCa diagnosis (64 years).

Regarding PCa treatment, several studies reported that radical prostatectomy may be considered a safe procedure for RTR (15, 33–37).

Nevertheless, surgery in RTR might be challenging for several factors, including the distortion of normal tissue planes by prior retroperitoneal surgery and the pelvic location of the transplant kidney and ureter making them susceptible to damage (14, 15, 37, 38).

Despite these potential difficulties, series from several institutions have documented the feasibility of radical prostatectomy in renal transplant recipients (14, 15, 37–39) via laparoscopic, open retropubic, and perineal approaches. Robot-assisted RP (RARP) has been incorporated into mainstream urologic practice and is now the most common approach to prostatectomy in Italy. Polcari et al. suggest that transperitoneal RARP is a feasible approach to the treatment of localized prostate cancer in a select group of renal transplant recipients (15).

Moreover, due to the potential challenge of bilateral obturator iliac lymphectomy, some authors suggest to perform unilateral lymphectomy at the side of the graft (9, 14, 35, 39, 40).

However, in our experience, when indicated, obturator iliac lymphectomy was performed bilaterally without graft damage or other operative or post-operative complications. In the systemic review by Hevia et al. (9), surgery was performed in 262 patients (82.1%) whereas, in our study, primary treatment with radiotherapy was performed in 10 (50%) patients with 1 (5%) of these that performed salvage radiotherapy after surgery. In line with previously reported, we did not observe any difference in Cox regression analysis in terms of overall survival between patients who underwent surgery and those who were treated with radiotherapy. Screening for and treatment of PCa in applicants for KT or in patients after KT should, therefore, be performed in an individualized manner on the basis of lifetime risk calculations.

Notably, a non-negligible option in PCa treatment -especially in the last years- is represented by AS. As reported by Aminsharifi et al. Current trends in renal transplantation, such as improved allograft/recipient survival and expanded organ transplantation eligibility criteria in older recipients, are concomitant with increasingly detected low risk prostate cancer in candidates for or recipients of renal transplantation (41). Thus, the role of AS protocol in RTR and the incorporation of modern imaging modalities to better select active surveillance candidates remain a clinical unmet need which may prevent overtreatment in such complex cases. In our case series, we recorded eight patients with a low risk PCa. At the time of decision making, the patients were scheduled for an active treatment, given the absence of robust AS protocols in RTR. In the era of PCa stage migration to low risk disease and given the absence of evidence on detrimental effect of KT in patients with low risk disease, AS might have been a valid option in this cases at this time.

Strengths of our study are represented by: (a) the opportunity to offer different active treatment options in patients with PCa and KT; in fact, thanks to a large experience in both urological and transplant surgery we overcame the potential difficulties in this type of surgery combined with the potential frailty of this patients which might have addressed the decision making to non-surgical options; (b) a long follow-up period which allowed us to evaluate oncological outcomes of different active options; (c) the opportunity to manage PCa after KT as any non-organ transplant patients, with the same range of therapeutic options.

Despite the homogeneous case series of RTRs with PCa analyzed, our study is not avoid of limitations: firstly, the retrospective nature of the data with a small sample size, which precludes the opportunity for definitive conclusions; secondly, a very large timeframe for the collection of data from patients with PCa after KT; in this regard, on the one hand a large period offer the opportunity to identify a greater number of patients for a relatively rare condition, on the other hand it exposes itself to the risk of not considering possible new diagnostic and therapeutic opportunities and new technologies that have appeared in the urological panorama in recent years. For example, the increasing use of magnetic resonance imaging in recent years, which has now entered in urological routine clinical practice (13), fusion transrectal or transperineal biopsy, as well as therapeutic opportunities such as focal therapy (cryotherapy, HIFU, etc.), as well as Intensity modulated radiation therapy (IMRT) or brachytherapy, were not considered by this study as alternative options and they were not included in the profile of diagnosis and treatment at the time of decision making process. Thirdly, the majority of cases in our series were well-differentiated and localized tumors at early stage (so that the treatment groups were nor formed or grouped according to any particular characteristics). Fourthly, the choice for unilateral vs. bilateral lymphadenectomy was based on surgeon's preference and this might potentially limit the overall reproducibility of the results. Lastly, the lack of complete information concerning survival outcomes, clinical PCa characteristics and PCa specific mortality.

According to our experience, surgical management of PCa as well as external radiotherapy in RTRs can be safely performed with favorable postoperative and oncological outcomes. Further studies are needed to explore benefits and harms of different therapeutic strategies for PCa in RTRs as well as to clarify which are the real indications for treatment strategies in the post-transplant population according to both tumors and patients' characteristics.

## Data Availability Statement

The original contributions presented in the study are included in the article/supplementary material, further inquiries can be directed to the corresponding author/s.

## Ethics Statement

The studies involving human participants were reviewed and approved by Comitato Etico Area Vasta Centro. The patients/participants provided their written informed consent to participate in this study.

## Author Contributions

DV and SCG: conception and design. FS and CS: statistical analysis. DV and CS: analysis and interpretation of data. CB, PP, AR, LV, DB, and MZ: acquisition of data. FS, GR, and PS: drafting of the manuscript. GV, GNe, GNi, and SS: critical revision of the manuscript. SS: supervision. All authors contributed to the article and approved the submitted version.

## Conflict of Interest

The authors declare that the research was conducted in the absence of any commercial or financial relationships that could be construed as a potential conflict of interest. The reviewer JGR declared a past co-authorship with the authors FS and SS to the handling editor.

## Publisher's Note

All claims expressed in this article are solely those of the authors and do not necessarily represent those of their affiliated organizations, or those of the publisher, the editors and the reviewers. Any product that may be evaluated in this article, or claim that may be made by its manufacturer, is not guaranteed or endorsed by the publisher.
